# Occurrence and Antigenic Specificity of Perinuclear Anti-Neutrophil Cytoplasmic Antibodies (P-ANCA) in Systemic Autoimmune Diseases

**DOI:** 10.3390/cells10082128

**Published:** 2021-08-19

**Authors:** Ourania D. Argyropoulou, Andreas V. Goules, Georgios Boutzios, Alexandra Tsirogianni, Charalampos Sfontouris, Menelaos N. Manoussakis, Panayiotis G. Vlachoyiannopoulos, Athanasios G. Tzioufas, Efstathia K. Kapsogeorgou

**Affiliations:** 1 Department of Pathophysiology, School of Medicine, National and Kapodistrian University of Athens, 11527 Athens, Greece; oargyrop@med.uoa.gr (O.D.A.); gboutzios@gmail.com (G.B.); menman@med.uoa.gr (M.N.M.); pvlah@med.uoa.gr (P.G.V.); agtzi@med.uoa.gr (A.G.T.); ekapso@med.uoa.gr (E.K.K.); 2 Joint Rheumatology Academic Program, School of Medicine, National and Kapodistrian University of Athens, 11527 Athens, Greece; 3 Department of Immunology and Histocompatibility, Evangelismos General Hospital, 10676 Athens, Greece; alextsir@gmail.com; 4 Department of Rheumatology, Evangelismos General Hospital, 10676 Athens, Greece; chsfont@yahoo.com

**Keywords:** P-ANCA autoantibodies, myeloperoxidase, elastase, vasculitis, systemic autoimmune rheumatic diseases

## Abstract

Perinuclear anti-neutrophilic cytoplasmic antibodies (P-ANCA) recognize heterogeneous antigens, including myeloperoxidase (MPO), lactoferrin, elastase, cathepsin-G and bactericidal/permeability-increasing protein. Although P-ANCA have diagnostic utility in vasculitides, they may also be found in patients with various other systemic autoimmune rheumatic diseases (SARDs). Nevertheless, the clinical significance and the targets recognized by P-ANCA in such patients remain unclear. For this purpose, herein we investigated the occurrence of ANCA-related antigenic specificities in 82 P-ANCA-positive sera by multiplex ELISA, as well as their association with other autoantibodies. The P-ANCA-positive sera corresponded to patients with vasculitides (*n* = 24), systemic lupus erythematosus (*n* = 28), antiphospholipid syndrome (*n* = 5), Sjögren’s syndrome (*n* = 7), rheumatoid arthritis (*n* = 3), systemic scleroderma (*n* = 1), sarcoidosis (*n* = 1) and Hashimoto′s thyroiditis (*n* = 13). In most P-ANCA-positive patients studied (51/82, 62.3%), these autoantibodies occurred in high titers (>1:160). The analysis of P-ANCA-positive sera revealed reactivity to MPO in only 50% of patients with vasculitides, whereas it was infrequent in the other disease groups studied. Reactivity to other P-ANCA-related autoantigens was also rarely detected. Our findings support that high P-ANCA titers occur in SARD. The P-ANCA-positive staining pattern is associated with MPO specificity in vasculitides, while in other autoimmune diseases, it mostly involves unknown autoantigens.

## 1. Introduction

Anti-neutrophil cytoplasmic antibodies (ANCA) are autoantibodies, mainly of IgG isotype, directed against proteins in the cytoplasmic granules of neutrophils and lysosomal proteins of monocytes. Depending on their staining pattern on alcohol-fixed neutrophils, ANCA are classified as diffuse cytoplasmic (C-ANCA), perinuclear (P-ANCA) and atypical (A-ANCA), the first two being highly significant for the diagnosis of ANCA-associated vasculitides. Myeloperoxidase (MPO) represents the major autoantigen recognized by P-ANCA, followed by neutrophil elastase, lactoferrin, cathepsin G, bactericidal/permeability-increasing protein (BPI), catalase and lysozyme, among others [1]. C-ANCA targeting proteinase-3 (PR3) has been associated with granulomatosis with polyangiitis (GPA), whereas P-ANCA targeting MPO is associated with microscopic polyangiitis (MPA). Patients with vasculitis and P-ANCA targeting MPO are most likely suffering from MPA (55–65%), followed by eosinophilic granulomatosis with polyangiitis (EGPA) (30–40%) and GPA (20–30%) [2]. Emerging evidence suggests that ANCA specificity associates with disease activity and may affect the clinical phenotype, as well as response to treatment, risk of relapse and long-term prognosis. To this end, MPA patients with MPO-ANCAs are more likely to develop isolated crescentic glomerulonephritis [3,4], pulmonary fibrosis and peripheral neuropathy [5,6], while MPO+-GPA patients have more frequently limited disease, without severe organ involvement, less need for cyclophosphamide or rituximab therapy and fewer relapses than those with proteinase-3 (PR3)-ANCA [7,8]. Interestingly, reappearance of MPO-ANCAs indicates relapse in more than 75% of patients [9].

Beyond MPA, P-ANCA have been described in a variety of other systemic autoimmune rheumatic diseases (SARDs), as well as chronic infections [10]. Indeed, MPO-ANCAs have been reported in systemic lupus erythematosus (SLE; 9.3%) [11], rheumatoid arthritis (RA; 4–18%) [12], Sjögren’s syndrome (SS; <3%) [13] and systemic sclerosis (SScl; <2.4%) [14]. Their presence has been associated with vasculitic patterns of glomerulonephritis and/or pulmonary involvement, while other P-ANCA-specific autoantigens, such as lactoferrin, neutrophil elastase, cathepsin and lysozyme, have also been described, although without known clinical significance [13,15,16]. In this context, P-ANCA and their distinct targets may have a potential role in distinguishing clinical phenotypes, disease prognosis and/or treatment monitoring. The aim of this study was to investigate the occurrence and the autoantigenic targets recognized by P-ANCA in various SARDs.

## 2. Materials and Methods

### 2.1. Patients’ Characteristics

The sera that have been examined for ANCA positivity by indirect immunofluorescence (IIF) in two highly experienced Greek diagnostic immunology laboratories (Department of Pathophysiology, School of Medicine, National and Kapodistrian University of Athens—a laboratory participating in the annual European Consensus Finding Study (ECFS) for Autoantibodies in Rheumatic Diseases in the context of EULAR and the Department of Immunology and Histocompatibility, Evangelismos General Hospital, Athens, Greece) during the past two years have been included in the study. From a total of 550 patients who were evaluated, 82 were found to be positive for the presence of P-ANCA by IIF and were included in the study. The medical records of all P-ANCA(+) patients were retrospectively analyzed and cumulative clinical, laboratory and autoantibody profile data were collected. Patients were classified into various systemic autoimmune diseases based on international classification criteria [15,17,18,19,20,21,22,23,24,25,26,27]. Subgroup analysis to identify clinical associations with P-ANCA autoantibodies was performed in terms of P-ANCA titers, type of autoimmune disease and comparison with control patients whenever applicable. The study was approved by the Ethics Committee of School of Medicine, National and Kapodistrian University of Athens, Greece (protocol no: 1718016656), following the general data protection regulations (GDPR) of European Union and the Helsinki Declaration principles. All sera samples were stored at −20 °C immediately after sampling and kept there until use.

### 2.2. Detection of P-ANCA Specificity and Associated Antigen Reactivity in Serum

The presence and titer of P-ANCAs were evaluated by standard IIF analysis on alcohol-fixed neutrophils using the NOVA Lite ANCA kit, Inova Diagnostics Inc. (San Diego, CA, USA) according to the manufacturer’s instructions, followed by evaluation of the staining pattern by fluorescence microscopy. Positive sera at a dilution of 1:20 (positive cut-off threshold) were serially diluted until becoming negative and the last positive dilution was considered as the P-ANCA titer. The antigens recognized by P-ANCA were further evaluated by a commercially available multiplex ELISA (ANCA profile ELISA, Euroimmun, Lubeck, Germany), analyzing the reactivity against MPO, lactoferrin, neutrophil elastase, cathepsin G and BPI antigens, semi-quantitatively and according to the manufacturer’s instructions.

### 2.3. Detection of Anti-Nuclear (ANA) and Other Autoantibodies in Serum

Similar to ANCA detection, the presence and titer of ANA were evaluated by IIF using the NOVA Lite HEp-2 ANA kit, Inova Diagnostics Inc in serial serum dilutions starting from 1:160 dilution and fluorescence microscopy. Anti-Ro(SSA), anti-La(SSB), anti-Sm, anti-U1RNP, anti-Scl70 and anti-RibP(IgG) autoantibodies were tested by immunoblotting using the Euroline Anti-ENA ProfilePlus1 (IgG) and Euroline ANA Profile-3 kits, Euroimmun. The levels of IgG and IgM antibodies against cardiolipin (aCL), β2GPI and double-stranded DNA (ds-DNA) were determined by home-made ELISAs, as previously described [28,29,30,31]. Anti-CCP, anti-TPO and anti-Tg were measured by commercially available ELISAs (QUANTA Lite CCP3.1 IgG/IgA, QUANTA Lite TPO and QUANTA Lite Thyroid T ELISA kits, Inova Diagnostics Inc.) according to the manufacturer’s instructions, whereas rheumatoid factor (RF) was detected by agglutination assay using commercially available latex-based reagents (RapiTex RF, Siemens Healthcare Diagnostics Products GmbH, Marburg, Germany) according to manufacturer’s instructions.

### 2.4. Statistics

Comparisons of categorical data were performed by chi square or Fisher exact test when cell counts were <5. For continuous variables, the Shapiro–Wilk normality test was performed initially, followed by the Mann–Whitney–Wilcoxon or *t*-test accordingly. Statistical analyses were performed using Python 3.6, and R software 4.0.3.

## 3. Results

### 3.1. Patients’ Characteristics

Eighty two of 550 tested patients were found to be positive for P-ANCA autoantibodies (in titer ≥1:20 dilution), 69 (84.2%) of whom fulfilled the criteria of a systemic autoimmune rheumatic disease (SARD) and 13 (15.9%) who presented with Hashimoto thyroiditis (HT). The 69 P-ANCA-positive SARD patients included 28 (40.6%) with systemic lupus erythematosus (SLE), 24 (34.8%) with a form of systemic vasculitis (18 with microscopic polyangiitis [MPA], two each with Behcet’s disease [BD] and Henoch–Schönlein purpura [HSP] and one each with aortitis and cryoglobulinemic vasculitis), 7 (10.2%) with Sjögren’s syndrome (SS), 5 (7.2%) with primary antiphospholipid syndrome (APS), 3 (4.4%) with rheumatoid arthritis (RA), and one each with systemic sclerosis (SSCL) and sarcoidosis.

The majority of SARD patients (57/69, 82.6%), as well as HT patients (11/13, 84.6%) were women. The median age at the time of P-ANCA measurement was 58 years (range: 20–85) for the SARD group and 55 years (range: 34–77) for those with HT patients. A more detailed description of patients’ characteristics per disease group is presented in Supplementary Tables S1 and S2.

### 3.2. P-ANCA Titers and Serum Autoantigen Specificity Per Autoimmune Disease

**Figure 1:**
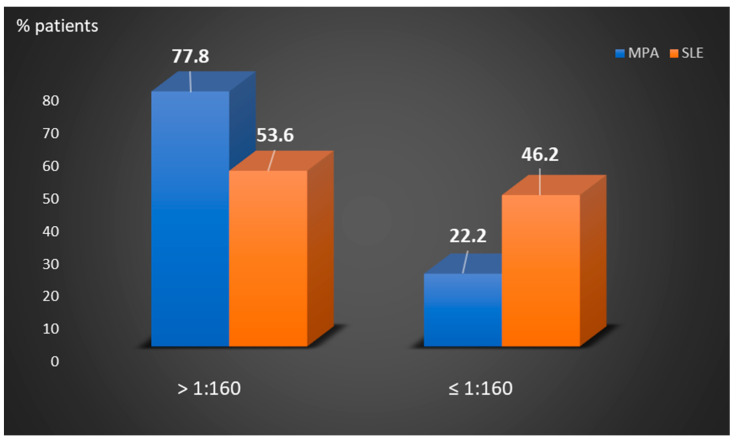
Comparison of the percentages of P-ANCA-positive MPA and SLE patients according to low ≤1/160 or high >1/160 titers. MPA: microscopic polyangiitis, SLE: systemic lupus erythematosus.

As detected by IIF, the titers of P-ANCA autoantibodies in the 82 P-ANCA-positive sera ranged from 1:20 to 1:640 (median: 640, Table 1). The majority of patients with SARD who were studied (50/69, 72.5%) presented with high P-ANCA-titer, namely, ≥1:80 (in 50/69, 72.5%) or ≥1:160 (in 43/69, 62.3%). Microscopic polyangiitis patients had higher P-ANCA titers compared to SLE patients (Figure 1). Among the 18 sera of P-ANCA-positive MPA patients, 11 (61.1%) presented reactivity to MPO (MPA-P-ANCA-MPO-positive), whereas the remaining seven did not exhibit any reactivity against the various autoantigens examined (MPA-P-ANCA-NS). On the other hand, the vast majority of the patients with other SARD were not found to recognize any of the P-ANCA-related antigens under investigation, including 25/28 (89.3%) of SLE patients studied. In fact, monospecific P-ANCA-positive patient cases with anti-MPO reactivity included one each with SLE, APS, RA and systemic sclerosis, whereas one patient with SS reacted with elastase and a SLE patient with lactoferrin. In addition, a SLE patient had double specificity for MPO/lactoferrin (Table 2).

### 3.3. Autoantibody Profile of P-ANCA Positive Patients and P-ANCA Related Specificity

**Figure 2:**
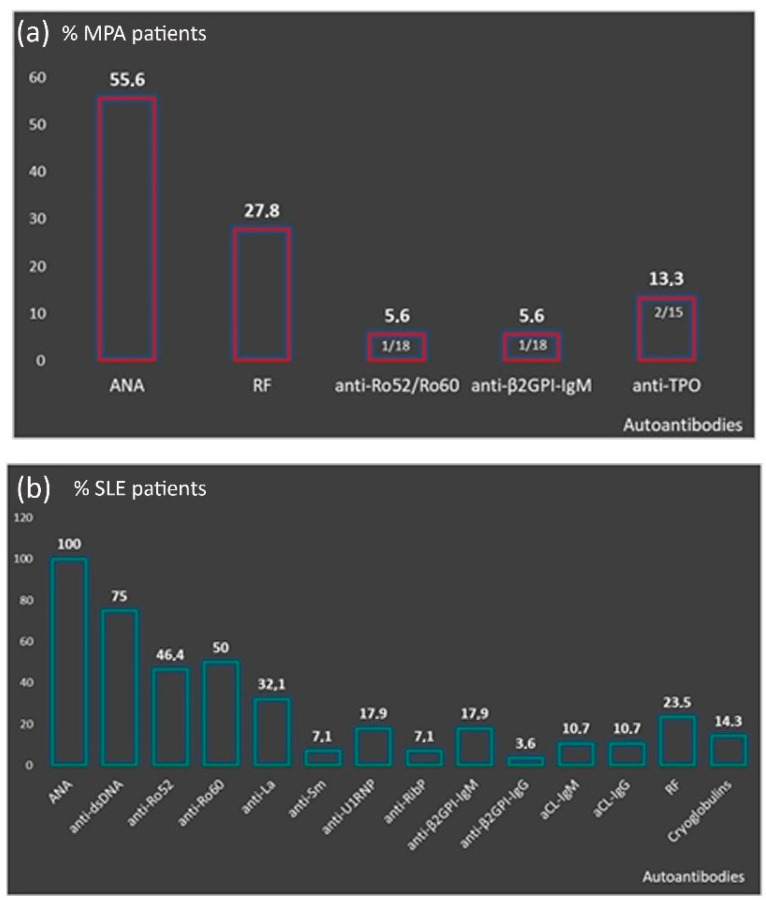
Autoantibody profile of P-ANCA positive patients. (**a**) Presence other than P-ANCA autoantibodies in MPA patients; (**b**) distribution of autoantibodies in the context of SLE. P-ANCA: perinuclear antineutrophil cytoplasmic antibodies, MPA: microscopic polyangiitis, SLE: systemic lupus erythematosus, ANA: antinuclear antibodies, RF: rheumatoid factor, aCL: anti-cardiolipin.

Microscopic polyangiitis-P-ANCA patients had increased frequency of ANA with a titer ranging from 1:160 to 1:1280 [1:160: 30%, *n* = 3/10, 1:320: 50%, *n* = 5/10, 1:640: 10%, *n* = 1/10 and 1:1280: 10%, *n* = 1/10) and RF (27.8%, *n* = 5/18) (Figure 2A). Compared to MPA-P-ANCA-NS, MPA-P-ANCA-MPO-positive patients had higher prevalence of ANA (63.6%, *n* = 7/11 vs. 42.9%, *n* = 3/7) and RF (36.4%, *n* = 4/11 vs. 14.3%, *n* = 1/7) and lower prevalence of anti-β2GPI-IgM (0% vs. 14.3%, *n* = 1/7), anti-Ro52/Ro60 (0% vs. 14.3%, *n* = 1/7) and anti-TPO (9,1%, *n* = 1/11 vs. 14.3%, *n* = 1/7), although these differences did not reach statistical significance (data not shown). The autoantibody profile of P-ANCA-positive SLE patients is presented in Figure 2B. All were ANA positive (28/28), with the vast majority having anti-dsDNA antibodies (in 21/25, 75%) and anti-Ro60/SSA autoantibodies (in 14/28, 50%). The majority of P-ANCA-positive SS patients were ANA positive (5/7, 71.4%) and approximately half of them had also anti-Ro52/Ro60 autoantibodies (3/7, 42.9%). A detailed description of the autoantibody profile of the remaining SARD, as well as HT patients, is presented in Supplementary Table S3.

### 3.4. Disease Features of P-ANCA Positive Patients

The small size of each disease group hampered the reliable statistical analysis of possible associations between P-ANCA-reactivity and clinical phenotypes. However, it seems that the clinical features of P-ANCA-positive patients fall within the typical disease clinical spectrum. More analytically, MPA-P-ANCA patients presented with non-specific clinical manifestations, including fatigue (14/18) and fever (12/18), as well as organ threatening disease, such as interstitial lung disease (ILD) and/or infiltrates (12/18) and glomerulonephritis (11/18). The most frequent clinical manifestations of SLE-P-ANCA patents were skin rash and/or photosensitivity (22/28), arthralgia/arthritis (18/28), anemia of chronic disease (13/28) and renal involvement (9/28). Furthermore, the three SLE patients recognizing P-ANCA-related antigens presented with different clinical phenotype. The anti-MPO-positive patient suffered from fatigue, fever, arthralgia/arthritis, photosensitivity and hematological findings, including anemia, leukopenia and thrombocytopenia; the anti-lactoferrin-positive SLE patient had more severe disease with non-specific manifestations (fatigue, fever, sicca symptoms), inflammatory arthritis, skin rash/photosensitivity, lymphadenopathy, serositis, Libman-Sacks endocarditis, enteritis, anemia and leukopenia, while the SLE patient with the double anti-MPO/lactoferrin specificity presented with fatigue, sicca manifestations, arthralgias, skin rash/photosensitivity and hematological findings (anemia, leukopenia and thrombocytopenia). The SS-P-ANCA+ patients all had sicca symptoms, musculoskeletal manifestations (6/7), Raynaud’s phenomenon (3/7) and anemia of chronic disease (3/7). The anti-elastase-positive SS patient had sicca manifestations, lymphadenopathy and ILD. The P-ANCA-positive APS patients had major vascular events, including pulmonary emboli, and thrombocytopenia, while the anti-MPO/elastase patient had lupus-like phenotype with mouth ulcers, alopecia, Raynaud’s phenomenon, pulmonary emboli, leukopenia and thrombocytopenia. The SSCL-anti-MPO+ patient developed acute renal failure. The clinical and laboratory features of all P-ANCA(+) patients included in the study are summarized in Supplementary Tables S1 and S2.

## 4. Discussion

In this report, we investigated the occurrence of P-ANCA autoantibodies and their specificity in various autoimmune diseases. In accordance with previous studies [2], this study further indicates that MPO is the predominant autoantigen targeted by P-ANCA in MPA patients, whereas reactivity to other P-ANCA-related autoantigens, such as lactoferrin, may only sporadically be observed [32]. Nevertheless, our study indicates that the antigenic specificity of P-ANCA autoantibodies remains elusive in a significant proportion of such patients. On the other hand, our results support that high titers of P-ANCA autoantibodies are frequently observed in patients with systemic autoimmune diseases, other than MPA. Interestingly, despite the relatively high P-ANCA titers, the vast majority of these patient groups have unidentified specificities. In line with previous reports [4,11,12,16], the most frequently recognized antigen in P-ANCA-positive patients is MPO, whereas reactivity against other P-ANCA-related autoantigens, such as lactoferrin and elastase, were rarely observed. Importantly, P-ANCA-positive SLE and MPA patients presented with enriched autoantibody profile, implying systemic autoimmune responses against ubiquitous self-antigens. Although the number of SLE P-ANCA positive patients is small, it is noteworthy that P-ANCA positivity among these patients is associated with skin, renal and hematologic manifestations. Interestingly, the anti-MPO positive lupus patient had no renal involvement, while anti-lactoferrin specificity was linked to the most severe clinical phenotype as opposed to double anti-MPO/lactoferrin specificity relayed to a milder clinical picture. These findings imply that P-ANCA specificity may have clinical significance; however, further multicentric studies of large patient cohorts are needed to verify these observations.

Important limitations of this study were the small number of patients, as well as the small number of autoantigens included in the assay we applied. A variety of potential self-antigens may be recognized by P-ANCA autoantibodies. Thus, diverse autoantigen array and/or high throughput biotechnologies are required to reveal potentially hidden specificities of P-ANCA reactivity. On the other hand, although limited, the misinterpretation of A-ANCA, as P-ANCA, cannot be excluded since our detection was based on immunofluorescence detection of only ethanol-fixed neutrophils. The parallel use of ethanol and formaldehyde-fixed neutrophils for the detection of P-ANCA has been reported to discriminate false P-, atypical ANCA [33]. However, this approach applies mainly for recognizing P-ANCA targeting MPO, whereas it has been reported to be non-beneficial for the study of SARDs patients other than those with vasculitides [33,34,35]. Furthermore, the likelihood of the detection of P-ANCA-related pattern in the setting of concurrent high ANA titers cannot be excluded as ANA may produce indistinguishable immunofluorescent staining patterns on ethanol-fixed neutrophils. In fact, charge interactions between DNA and MPO may cause false positivity in MPO-ANCA in case of high anti-dsDNA sera titer [36]. However, this possibility is significantly diminished by the fact that the detection of the characteristic perinuclear ring pattern is a prerequisite finding for the diagnosis of P-ANCA staining pattern in our departments.

In summary, P-ANCA are present in sera of patients with various systemic autoimmune diseases in high titers and are associated with ANA, confirming to most likely be in support ofthe systemic nature of autoimmunity. The results of this study indicate that, although MPO represents the most common P-ANCA specificity for MPA, autoantibodies to additional novel neutrophilic self-antigens are likely present in P-ANCA-positive autoimmune disease patients. In this context, further investigation of the currently unidentified P-ANCA-related autoantigens may reveal novel and clinically useful disease markers.

## Figures and Tables

**Table 1:** Serum P-ANCA titers in the various P-ANCA-positive autoimmune disease patients studied.

Patient Groups		P-ANCA Serum Titers (No Positive)					
		≥1:640	1:320	1:160	1:80	1:40	1:20
Vasculitides	MPA (*n* = 18)	9	5	1	1	1	1
	BD (*n* = 2)	1	1	0	0	0	0
	Aortitis (*n* = 1)	1	0	0	0	0	0
	HSP (*n* = 2)	0	0	1	1	0	0
	CV (*n* = 1)	0	1	0	0	0	0
SLE (*n* = 28)		9	6	3	7	23	0
APS (*n* = 5)		1	1	1	1	0	1
SS (*n* = 7)		5	0	0	0	0	2
RA (*n* = 3)		2	0	0	1	0	0
SSCL (*n* = 1)		0	1	0	0	0	0
Sarcoidosis	(*n* = 1)	0	0	1	0	0	0
Hashimoto	(*n* = 13)	2	1	4	2	1	3

P-ANCA: perinuclear antineutrophil cytoplasmic antibodies, MPA: microscopic polyangiitis, BD: Behcet’s disease, HSP: Henoch–Schonlein purpura, CV: cryoglobulinemic vasculitis, SLE: systemic lupus erythematosus, APS: antiphospholipid syndrome, SS: Sjögren’s syndrome, RA: rheumatoid arthritis, SSCL: systemic sclerosis.

**Table 2:** P-ANCA-related antigenic specificities in the various P-ANCA-positive autoimmune disease patients studied.

Autoimmune Diseases		Antigens Recognized by P-ANCA Positive Sera (No Positive)					
		MPO	Elastase	Cathepsin G	BPI	Lactoferrin	MPO/Lactoferrin
Vasculitides	MPA (*n* = 18)	**11**	0	0	0	0	0
	BD (*n* = 2)	**1**	0	0	0	0	0
	Aortitis (*n* = 1)	0	0	0	0	0	0
	HSP (*n* = 2)	0	0	0	0	0	0
	CV (*n* = 1)	0	0	0	0	**1/1 (100)**	0
SLE (*n* = 28)		**1**	0	0	0	**1/28 (3.6)**	**1/28 (3.6)**
APS (*n* = 5)		**1**	0	0	0	0	0
SS (*n* = 7)		0	**1**	0	0	0	0
RA (*n* = 3)		**1**	0	0	0	0	0
SSCL (*n* = 2)		**1**	0	0	0	0	0
Sarcoidosis (*n* = 1)		0	0	0	0	0	0
Hashimoto (*n* = 13)		0	0	0	0	0	0

P-ANCA: perinuclear antineutrophil cytoplasmic antibodies, MPO: myeloperoxidase, BPI: bactericidal/permeability-increasing protein, MPA: microscopic polyangiitis, BD: Behcet’s disease, HSP: Henoch–Schonlein purpura, CV: cryoglobulinemic vasculitis, SLE: systemic lupus erythematosus, APS: antiphospholipid syndrome, SS: Sjögren’s syndrome, RA: rheumatoid arthritis, SSCL: systemic sclerosis. Positive values are highlighted by bold type letters.

## Data Availability

The data will be available upon request.

## Supplementary Materials

The following are available online at https://www.mdpi.com/article/10.3390/cells10082128/s1, Table S1: Clinical and Laboratory features of patients with systemic vasculitis, SLE and APS, Table S2: Clinical and Laboratory features of patients with other autoimmune diseases, Table S3: Autoantibody profile of other than MPA and SLE pANCA positive patients sub-grouped per disease type.
