# Supplementary material for: Occurrence and Antigenic Specificity of Perinuclear Anti-Neutrophil Cytoplasmic Antibodies (P-ANCA) in Systemic Autoimmune Diseases
Source: Cells. 2021 Aug 19;10(8):2128. doi: 10.3390/cells10082128 (PMC8393570; doi:10.3390/cells10082128)
Supplement: Supplementary file 1 [file cells-10-02128-s001.zip › cells-1335405-supplementary.pdf]

Supplementary Table S1. Clinical and Laboratory features of patients with systemic vasculitis, SLE and APS.

	Vasculitis patients (n=24)					LE/APS patients (n=33)	
	MPA (n=18)	BD (n=2)	Aortitis (n=1)	HSP (n=2)	CV (n=1)	SLE (n=28)	APS (n=5)
Age (Median years, range)	65 (27-85)	47(42-52)	73	47(17-59)	77	47.5 (20-72)	43(31-73)
Gender (men/women)	3/15	1/1	0/1	2/0	0/1	4/24	1/4
Clinical & laboratory features *(No of Patients)*							
Non-specific							
Chronic fatigue	14	1	1	0	1	11	0
Fever	12	1	1	2	1	4	1
SICCA	2	0	1	2	1	6	1
Glandular							
SGE	0	0	0	0	0	2	0
Lymphadenopathy	4	0	0	0	0	3	0
Eye involvement	1	0	0	0	0	0	0
Serositis	2	0	0	0	0	4	0
Musculoskeletal involvement	8	1	1	2	1	18	2
ILD	6	0	0	0	0	0	0
Infiltrates/Hemoptisis	8	0	0	0	0	0	0
Glomerulonephritis	10	0	0	0	0	9	0
IgA nephropathy	0	0	0	1	0	0	0
PNS	2	0	0	0	1	0	0
CNS	0	0	0	0	0	2	0
Skin involvement							
Purpura	1	1	0	2	1	2	0
Mucosal ulcers	0	2	0	1	0	0	1
Other	2	1	0	0	0	22	2
Vascular							
Raynaud's phenomenon	0	0	0	0	1	3	1
Vasculitic ulcers	0	0	0	0	0	0	0
DVT	2	0	0	0	0	2	0
PE	1	0	0	0	0	0	3
Aneurysms	0	0	1	0	0	0	0
Stroke	0	0	0	0	0	0	1
Cardiac	0	0	0	0	0	1	0
Gastrointestinal inv	0	0	0	1	0	1	0
Laboratory features							
Anemia of chronic disease	16	1	1	1	0	13	1
AHA	0	0	0	0	0	1	1
Leukopenia	0	0	0	0	0	6	1
Thrombocytopenia	0	0	0	0	0	2	3

MPA: microscopic polyangiitis, BD: Behcet's disease, HSP: purpura Henoch Schonlein, CV: cryoglobulinemic vasculitis, SLE: systemic lupus erythematosus, APS: antiphospholipid syndrome, SGE: salivary gland enlargement, ILD: interstitial lung disease, PNS: peripheral nervous system, CNS: central nervous system, DVT: deep venous thrombosis, PE: pulmonary emboli, inv: involvement, AHA: autoimmune hemolytic anemia.

Supplementary Table S2. Clinical and Laboratory features of patients with other autoimmune diseases.

	SS (n=7)	RA (n=3)	SSCL (n=1)	Thyroiditis Hashimoto (n=13)	Sarcoidosis (n=1)
**Age [Median (years), range]**	74(40-80)	50(39-64)	64	55(34-77)	45
**Gender (man/woman)**	1/6	0/3	0/1	2/11	0/1
**Clinical & laboratory features** (Patient No)					
**Non-specific**					
Chronic fatigue	3	1	1	2	1
Fever	2	0	0	0	0
Sicca	7	2	0	0	0
**Glandular**					
SGE	1	0	0	0	1
Lymphadenopathy	1	1	0	0	1
**Eye involvement**	0	1	0	0	0
**Serositis**	1	1	0	0	0
**Musculoskeletal involvement**	6	3	1	7	0
**ILD**	1	0	0	0	0
**Infiltrates/Hemoptisis**	0	0	0	0	0
**Glomerulonephritis**	0	0	0	0	0
**IgA nephropathy**	0	0	0	0	0
**PNS**	0	0	0	0	0
**CNS**	0	0	0	0	0
**Skin involvement**					
Purpura	2	0	0	0	0
Mucosal ulcers	0	0)	0	1	0
Other	1	0	1	2	0
**Vascular**					
Raynaud's phenomenon	3	0	0	1	0
Vasculitic ulcers	0	0	0	0	0
DVT	0	0	0	0	0
PE	0	0	0	0	0
Aneurysms	0	0	0	0	0
Stroke	0	0	0	0	0
**Cardiac**	0	0	0	0	0
**Gastrointestinal inv**	0	0	0	1	0
	0	0	0	0	0
**Laboratory features**					
Anemia of chronic disease	3	0	0	1	0
AHA	0	0	0	0	0
Leukopenia	1	0	0	5	0
Thrombocytopenia	0	0	0	0	0

SS: Sjogren's syndrome, RA: rheumatoid arthritis, SSCL: systemic sclerosis, SGE: salivary gland enlargement, ILD: interstitial lung disease, PNS: peripheral nervous system, CNS: central nervous system, DVT: deep venous thrombosis, PE: pulmonary emboli, inv: involvement, AHA: autoimmune hemolytic anemia.

Supplementary Table S3. Autoantibody profile of other than MPA and SLE pANCA positive patients sub-grouped per disease type.

Antibodies % (n)	Vasculitides (n=24)				APS (n=5)	SS (n=7)	RA (n=3)	SSCL (n=1)	Thyroiditis Hashimoto (n=13)	Sarcoidosis (n=1)
	BD (n=2)	Aortitis (n=1)	HSP (n=2)	CV (n=1)						
**ANA**	**1**	**1**	**1**	**1**	**1**	**5**	**2**	**1**	**4**	**1**
**dsDNA**	**1**	0	0	0	0	0	0	0	0	0
**Ro52**	0	**1**	0	**1**	0	**3**	0	0	**1**	0
**Ro60**	0	**1**	0	**1**	0	**3**	0	0	**1**	0
**Ro52/Ro60**	0	**1**	0	**1**	0	**3**	0	0	**1**	0
**La**	0	0	0	0	0	**2**	0	0	0	0
**SM**	0	0	0	0	0	**1**	0	0	0	0
**UIRNP**	0	0	0	0	**1**	**1**	0	0	0	0
**scl70**	0	0	0	0	0	0	0	0	0	0
**RibP**	NM	NM	NM	NM	NM	NM	NM	NM	NM	NM
**aCL-IgM**	**1**	0	0	0	**2**	**2**	0	0	0	0
**aCL-IgG**	0	0	0	0	**1**	0	0	0	0	0
**β2GPI-IgM**	**1**	0	0	0	**2**	**1**	**1**	0	0	0
**β2GPI-IgG**	0	0	0	0	**1**	0	**1**	0	0	0
**RF**	0	0	**1**	**1**	0	**2**	**2**	0	0	0
**Cryos**	0	0	0	**1**	NM	0	0	NM	0	0
**CCP**	0	0	0	0	0	**1**	**3**	NM	0	0
**TPO**	0	0	0	NM	**1**	**3**	0	NM	**9**	0
**TG**	0	0	0	NM	**1**	0	0	NM	**11**	0

pANCA: perinuclear antineutrophil cytoplasmic antibodies, MPA: microscopic polyangiitis, BD: Behcet's disease, HSP: Henoch-Schonlein purpura, CV: cryoglobulinemic vasculitis, SLE: systemic lupus erythematosus, APS: antiphospholipid syndrome, SS: Sjögren's syndrome, RA: rheumatoid arthritis, SSCL: systemic sclerosis, ANA: antinuclear antibodies, RF: rheumatoid factor, aCL: anti-cardiolipin, cryos: cryoglobulins.
